# P-1703. Evaluation of Antibiotic Use for Dental Infections in Dental Clinics Associated with an Academic Safety Net Institution

**DOI:** 10.1093/ofid/ofae631.1869

**Published:** 2025-01-29

**Authors:** Michael A Deaney, Margaret M Cooper, Timothy C Jenkins, Kimberly A Meyers, Katherine C Shihadeh

**Affiliations:** Denver Health Medical Center, Denver, Colorado; Denver Health Medical Center, Denver, Colorado; Denver Health, Denver, Colorado; Denver Health Medical Center, Denver, Colorado; Denver Health, Denver, Colorado

## Abstract

**Background:**

In 2019, the American Dental Association (ADA) issued guidance on diagnostic criteria and appropriate antibiotic regimens for dental pain and swelling. Limited data exists on adherence to these guidelines. This study aimed to evaluate adherence to these guidelines within outpatient dental clinics.

Figure 1

**Figure:**
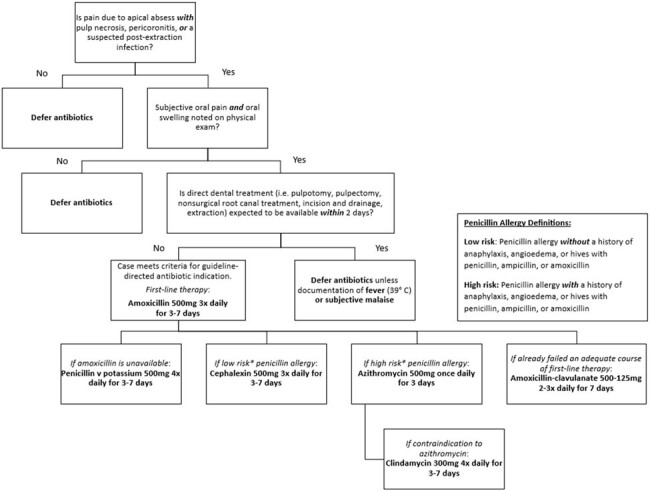

A flowchart for assessing antibiotic indication, selection, dose and duration adapted from the 2019 American Dental Association (ADA) guideline on antibiotic use for the urgent management of pulpal- and periapical-related dental pain and intraoral swelling

**Methods:**

We electronically identified all antibiotic prescriptions written by dentists in seven outpatient dental clinics in Denver Health, an integrated academic safety net institution, from July 2022 to November 2023. Detailed medical record review was performed for 200 randomly selected patients who were prescribed an antibiotic to examine antibiotic indication, selection, dose, and duration. Assessment of whether the prescribed regimens adhered to guidelines was conducted using a flowchart adapted from the ADA guidance (Figure 1).

**Results:**

Forty-nine dentists dispensed a total of 1,874 prescriptions at 114,231 clinic visits (1.6% of visits). In the 200 patients whose medical records were reviewed, common prescription indications were irreversible pulpitis (15.8%), pulp necrosis with apical abscess (14.3%), caries (14.3%), and concern for post-operative infection (12.8%). Pain was noted in 85.2% of cases, with a median pain scale score of six. Due to incomplete documentation, 46.9% of prescriptions were deemed as "indeterminate" for guideline-directed indication. Only 5.6% of the documented cases met full indication criteria for antibiotic use per ADA guidance. Antibiotic selection was consistent with ADA guidance in 81.6% of cases; amoxicillin was the most prescribed antibiotic (79% of cases), followed by clindamycin (9.5%) and amoxicillin-clavulanate (8%). Durations of therapy were longer than recommended in ADA guidance in 23% of cases. In total, only 3.1% of prescriptions adhered to ADA guideline-directed antibiotic indication, selection, dose, and duration.

**Conclusion:**

An antibiotic was prescribed in < 2% of dental clinic visits; while this rate aligns with previous literature, there is opportunity to improve adherence to ADA guideline-directed therapy for dental infections. Evaluation of effective stewardship interventions reinforcing these guidelines is warranted.

**Disclosures:**

**All Authors**: No reported disclosures

